# “But It’s Just a Fish”: Understanding the Challenges of Applying the 3Rs in Laboratory Aquariums in the UK

**DOI:** 10.3390/ani9121075

**Published:** 2019-12-03

**Authors:** Reuben Message, Beth Greenhough

**Affiliations:** School of Geography and the Environment, University of Oxford, Oxford OX1 2JD, UK; beth.greenhough@ouce.ox.ac.uk

**Keywords:** 3Rs, social science, qualitative analyses, fish, zebrafish, society-animal relations

## Abstract

**Simple Summary:**

Fish are widely used in research and some species have become important model organisms in the biosciences. Despite their importance, their welfare has usually been less of a focus of public interest or regulatory attention than the welfare of more familiar terrestrial and mammalian laboratory animals; indeed, the use of fish in experiments has often been viewed as ethically preferable or even neutral. Adopting a social science perspective and qualitative methodology to address stakeholder understandings of the problem of laboratory fish welfare, this paper examines the underlying social factors and drivers that influence thinking, priorities and implementation of fish welfare initiatives and the 3Rs (Replacement, Reduction and Refinement) for fish. Illustrating the case with original stakeholder interviews and experience of participant observation in zebrafish facilities, this paper explores some key social factors influencing the take up of the 3Rs in this context. Our findings suggest the relevance of factors including ambient cultural perceptions of fish, disagreements about the evidence on fish pain and suffering, the language of regulators, and the experiences of scientists and technologists who develop and put the 3Rs into practice. The discussion is focused on the UK context, although the main themes will be pertinent around the world.

**Abstract:**

Adopting a social science perspective and qualitative methodology on the problem of laboratory fish welfare, this paper examines some underlying social factors and drivers that influence thinking, priorities and implementation of fish welfare initiatives and the 3Rs (Replacement, Reduction and Refinement) for fish. Drawing on original qualitative interviews with stakeholders, animal technologists and scientists who work with fish—especially zebrafish—to illustrate the case, this paper explores some key social factors influencing the take up of the 3Rs in this context. Our findings suggest the relevance of factors including ambient cultural perceptions of fish, disagreements about the evidence on fish pain and suffering, the discourse of regulators, and the experiences of scientists and animal technologists who develop and put the 3Rs into practice. The discussion is focused on the UK context, although the main themes will be pertinent around the world.

## 1. Introduction

The relevance of human-animal interactions, relationships and bonds to laboratory animal welfare, robust animal-dependent science and ethics is widely acknowledged by practitioners, e.g., [1,2,3,4,5,6,7,8]. How these are embedded in and reflective of wider social processes, relations and structures is also increasingly a matter of interest to social scientists, historians and ethicists, many of whom are also concerned to better understand how such broader societal issues shape the implementation and development of public policy and associated ethical frameworks, e.g., [9,10,11], including the 3Rs [12,13]. There is also thriving literature on the role of public opinion concerning the use of laboratory animals, much of which illustrates an interest in how species differences can mediate social attitudes and potentially structure policy priorities, e.g., [14,15,16,17]. The case of the use of fish in regulated scientific research is a good example of this, but has seldom before been addressed for some partial exceptions, see [18,19,20,21]. Using the 3Rs as a point of entry, this paper adopts a qualitative social scientific perspective, highlighting social drivers that could be influencing thinking on, prioritization of and implementation of laboratory fish welfare.

In the United Kingdom and many other countries, fish have not historically qualified for sympathy because they were deemed too dissimilar to humans [22] (p. 177). Times have changed: following rising concerns about food fish sustainability, oceanic health, and the industrialization of both wild-capture fisheries and aquaculture, the ethics of human-fish relations in their different forms and locations have slowly become topics of both popular (e.g., [23,24,25,26]) and academic (e.g., [27,28,29,30,31,32]) criticism. Additionally, there has been an explosion of scientific interest in the cognitive abilities of fish and their capacity for emotional experiences, topics which tend to have a close association with debates about welfare, ethics and the controversy about fish pain, e.g., [33,34,35,36]. Fish welfare has also risen slowly up the agenda of animal welfare charities and campaign groups. Following the steep rise of finfish aquaculture in the global North, the websites of most of the large, multi-campaign issue organizations now feature dedicated pages to fish farming and humane slaughter. There are also a growing number of online campaign groups dedicated specifically to raising awareness about suffering in fisheries and advocating for fish sentience—Fish Feel, Let Fish Live, and fishcount.org are prominent examples see [37,38,39]. Via the European Union in particular, regulators have made attempts at entrenching the legal recognition of fish as sentient beings in practice, and have been active in areas including humane slaughter regulations and the harmonizing of husbandry standards for farmed fish, e.g., [40,41,42]. These and other developments (notably welfare-motivated restrictions on recreational angling in Switzerland and Germany) have recently led some fisheries biologists to wonder what the developing welfare agenda means for the future of aquaculture, angling, commercial fishing and research? [43]. 

However, the (re)emergence of discussions around contested moralities of recreational angling [44,45,46,47,48,49], welfare in the context of wild-capture fisheries [50,51,52,53], the ethics of dietary trends (pescetarianism) [54,55], and the putative demands amongst consumers in some countries for higher welfare farmed fish [56,57,58,59,60,61], all suggest that there remain stubborn, sometimes intractable, challenges in all of these areas. Growth in the number of commentators does not necessarily reflect serious changes in policy and practice. It is also not yet clear whether recent interest by the news media in scientific work exploring the mental and emotional capacities of some fish species—including for example their capacity to feel pain [62], pass the mirror test of putative self-awareness [63], or “pine” for their mates and get depressed [64,65]—either reflect or have provoked substantial changes in public attitude. What people make of such information is open to debate. The film Finding Nemo, with its positive and engaging portrayal of one the ocean’s most charismatic fish species and its famous line “fish are friends—not food”, was predicted to have caused a ripple effect in public sentiment towards fish and the aquatic world. However, ironically, when the geographer Driessen [66] investigated this, he discovered that the film had, in fact, become a popular name for cafés specializing in fish and chips. With one café already adopting it as a name, the same appears set to be true of Blue Planet II, David Attenborough’s hugely popular documentary, which has been credited with kick-starting debate about the state of the planet’s oceans, and showed the world footage of sophisticated and surprising fish behaviors, including tool use [67,68]. 

This wider social and cultural context is important when approaching the welfare situation of laboratory fish and the 3Rs. The intensification of fish use in laboratory research generally—and the rise to prominence of zebrafish models in particular—have likewise provoked higher levels of interest in the issue of fish welfare in the sector in the UK and internationally. This includes an emergence of reflections on different ethical issues associated with the use of fish in research specifically [21,69,70]. There has also been a growing willingness to consider, develop and implement 3Rs initiatives focused on fish amongst animal technologists, scientists, veterinarians and policy makers, and both the UK and pan-European laboratory animal welfare and veterinary organizations have all played different roles in highlighting fish welfare amongst their constituencies, e.g., [71,72,73]. Furthermore, there are direct links between developments in laboratory fish welfare and other sectors. In the UK, intensive aquaculture and the laboratory aquarium are connected via personnel, technology and knowledge transfer. For example, via links between forums such as the Fish Veterinary Society and the Laboratory Animal Veterinary Society (both subsections of the British Veterinary Society), or notable colloquiums organized by organizations like the National Centre for the Replacement, Refinement and Reduction of Animals in Research (NC3Rs) and the Centre for Environment, Fisheries and Aquaculture Science (CEFAS). 

Yet, again, there remains a widespread sense amongst those who work with fish or who regulate fish-based science that the degree of attention that fish of any species receive is not yet commensurate with the quantities in which they are used, their importance to science, nor—if much recent behavioral and neuroscientific evidence is accepted—their possible levels of suffering. As the authors’ have often heard in the course of their research, this sense is quite widely shared amongst scientists, technologists and others who work with fish (including zebrafish) in the UK. This can filter through and be reflected in efforts to prioritize 3Rs and other welfare-relevant interventions that benefit fish. By discussing challenges to the 3Rs with reference to wider context, this paper sets out to stimulate discussion and reflection by proposing that developments (or lack thereof) in this field are connected to a variety of interlinked social drivers, and scientific, institutional and regulatory viewpoints. 

## 2. Materials and Methods 

Within the social sciences, qualitative methods offer an effective and insightful means of understanding the intersection of the broader (largely utilitarian) ethical frameworks which shape animal research, and the more individualized moral convictions, beliefs and practices of those who work closely with laboratory animals and who are often tasked with implementing policy. Interviews and participant observation, alongside the analysis of key literatures, policy documents and archival materials, have formed the basis of several landmark studies in the field, e.g., [9,74], and have proved highly effective in developing understandings of how ethics and the 3Rs are “put into practice” in the field of animal research [75]. Adopting a similar approach, this paper seeks to energise debate on fish and the 3Rs by drawing on the authors’ experiences of participant observation in UK zebrafish facilities, participation in professional events and conferences, as well as interviews with stakeholders. It is not intended to be a technical review of 3Rs initiatives and related welfare issues for zebrafish (of these there are a growing number, see e.g., [21,69,76,77,78,79,80,81]). The objective here is to gain insights into how people who work with laboratory fish understand and explain their practices and their relationships to the humans and animals they work with, and also to the wider field of animal research. In other words, we are offering an account of the ways in which people talk about: (i) whether or not they (and others) care about fish (attitudes towards); and (ii) how this shapes their ability to care for them (husbandry practices). By relating these to wider literatures, policy documents and other textual sources we can begin to build up a picture of the key social norms and discourses in and around laboratory zebrafish research, the possible implications of these for fish welfare, and hopefully shed light on barriers to implementing and developing the 3Rs initiatives for from a sociological, rather than technical, point of view.

The arguments presented in this paper are derived from a larger body of ongoing research into the species and spaces of contemporary animal research in the UK, performed as a part of the collaborative research project “The Animal Research Nexus” project (see www.animalresearchnexus.org). This project seeks to understand the factors that have shaped and continue to underpin the social contract on which animal research in the UK rest, better understand emergent issues and challenges, and contribute positively to cultures of communication across the sector. This paper draws on data and insights developed in one sub-strand of this wider project. This strand of work focused on understanding the care and welfare work of animal technologists, the managers of aquarium facilities and scientists who work with fish, asking how their understandings of their work relate to wider ethical and legal frameworks. As a part of this, we also engaged with other stakeholders, including veterinary professionals and the regulators of animal research in the UK—in particular, Home Office Inspectors—as well as animal welfare organisations. 

This study adopts a mixed method approach, drawing on a combination of in-depth interviews, participant observation and documentary analyses. Firstly, in order to gain an insight into how fish welfare is put into practice, the first author has taken part in two one-week-long stints as a participant-observer in two different zebrafish aquariums in the UK, conducted repeat visits to a facility to see how they introduced a new zebrafish room, and participated in a professional training course for researchers and technicians who work with zebrafish. Secondly, we have reviewed publicly available documentation and relevant professional literature. Thirdly, we conducted in-depth semi-structured qualitative interviews with 27 individuals (two interviews involved more than one participant being interviewed at a time), including scientists, animal technologists, facility managers, veterinary professionals, representatives of animal welfare charities, and regulators. Additionally, both authors have paid shorter visits to numerous fish facilities across the UK over the past seven years, as well as attended and participating in a variety of professional conferences and related forums and engaging in ongoing collaboration and dialogue with the wider animal research community and associated stakeholders, including both those supportive of and against animal research. While we have interviewed three scientists based at contract research organizations and pharmaceutical companies, and those who specialise in commercial regulatory testing, our focus has been on university-based bioscience research. This is because this is where the vast majority of fish research in the UK is conducted. This is additionally justified because there is already a disproportionate focus on toxicology research in the 3Rs literature [82,83]. Lasting, on average, around one and a half hours, interviews were conducted, where possible, at the place of work of the interviewee. Interviews were digitally recorded, transcribed and analyzed thematically using the qualitative data analyses tool NVivo. A number of key themes were identified (Table 1). A close reading of the relevant sections of text associated with each of these codes was then used to establish which of these themes are most pertinent to understanding the social and cultural barriers to implementing the 3Rs for zebrafish welfare, the topic of this paper—other themes identified, of course, relate more to emergent elements of the wider program. This was justified with primary reference to what participants themselves said about the 3Rs, our own experience of interacting with stakeholders and working in zebrafish facilities (participant observation), reference to themes in associated literature (discourse analysis), as well as in the light of secondary social science literature on the social organisation of animal science and the 3Rs. 

While a number of foci suggest themselves, some of which the authors’ explore in forthcoming work, we have thus restricted the discussion below to three key themes: “knowledge and consensus”, “attitudes and experiences”, and “institutional support and capacity”.

In keeping with the intentions of qualitative research of this kind, emphasis is placed on depth as opposed to breadth. The sample size is small, and the results selected for presented here are indicative of a wide range of themes and key issues that should be taken into account rather than thought of as being representative in any way. A logical next step may be to use some of the perceived issues and concerns raised here as a basis for a larger, quantitative study. Inevitably, we have also neglected to discuss a number of important social and scientific issues relevant to understanding the challenges to taking up 3Rs initiatives focused on zebrafish, or fish in general. These include, for example, generic concerns about the lower status of animal welfare science and 3Rs related research versus the attractions of other fields of biological research and the relative ghettoisation of 3Rs research as a distinct category [84] (p.128). It is also possible that if and when concerns about the reproducibility of much zebrafish-based science grows, so too will “neophobia” increase in prominence as a barrier to 3Rs interventions with zebrafish though is not something reflected in our data [85].

Due to the sensitive nature of the topic (animal research), a policy of anonymisation and decontextualisation has been applied to all transcripts in order to ensure the privacy of participants. All names used in this paper are pseudonyms. All interviews were conducted with the written consent of participants. This research has been granted ethical approval by the Central University Research Ethics Committee (CUREC) of the University of Oxford (Reference Number: SOGE 18A-7). By agreement with the Wellcome Trust and research participants, anonymised interview transcripts will be deposited in the UK Data Archive based at the University of Essex (https://www.data-archive.ac.uk) after a period of 10 years from the completion of the Animal Research Nexus Project in 2022, except in cases where participants have explicitly opted out of this arrangement. 

### Focusing on Zebrafish

This paper focuses on zebrafish because, over the last three decades, they have become by far the most prominent species of fish used in animal research. In 2018, zebrafish accounted for 12 percent of all procedures done on live animals (including creation and breeding of GA lines) in the UK. All other species of fish combined accounted for 2.6 percent of animals used [86]. The species’ relatively steep rise towards the apex of lab “supermodels” has often meant that those seeking to develop 3Rs and other welfare-relevant scientific and husbandry protocols have had to work hard to keep up with the pace of change whilst striving to improve [80,87]. At the same time, the rise of the zebrafish, in conjunction with other trends such the intensification of aquaculture production and related public anxieties about environmental externalities and food safety, have most likely served to cast light onto the issue of fish welfare more generally, e.g., [42,88,89]. To this extent, and remaining mindful of the extensive diversity of fish kinds, many of the points made in this paper will nevertheless also be relevant to other fish species.

A number of factors are regularly cited as key attractions of the zebrafish model for biologists. These include its hardiness in captivity, small size, short generation time, rapid development and large clutch size. These factors also make them relatively cheap to maintain in large numbers. In addition, the comparative simplicity of the zebrafish genome facilitated the application of various molecular technologies. In combination with the extraordinary optical accessibility of its embryos and young larvae (they are transparent and fertilized externally to the mother’s body), these features have made zebrafish a highly tractable model for other vertebrate animals, and useful in a wide range of fields. However, these very advantages of the organism for science can also contribute to the entrenchment of particular attitudes towards them, and towards fish generally. Moreover, they can raise 3Rs considerations in their own right. Ironically, their hardiness in captivity has proven a disincentive for refining their husbandry conditions [90] (p.141). Depending on local aquarium practices and pricing structures, the low costs of maintaining zebrafish in large numbers and the ease with which they can usually be bred can create an incentive to keep transgenic lines running even when they are not being used, and a disincentive to cryopreserve and regenerate on demand—strategies which would be seen as more consistent with a reduction in animal use. The fact that zebrafish models can be valuable surrogates for other vertebrates also tends to compound the view of them as “lower” on the so-called phylogenetic scale, contributing in turn to the view that the use of fish (as embryos or larvae, but also adults) represents a kind of “relative replacement” for other vertebrate animals [91] (p.274), which is to say, a means for achieving 3Rs (replacement or refinement) targets, as opposed to individuals to whom the 3Rs principles of refinement, reduction and replacement could be applied (see also [92]).

The scale at which zebrafish are maintained and the ease and rate at which they can be induced to reproduce can also all contribute to a sense of their replaceability, and underline the difficulty of forming a bond with them as individuals—even in comparison to other small, short-lived and relatively easily replaced laboratory vertebrates like mice [93,94]. Some people, especially animal technologists, attempt to think of fish as unique individual beings that deserve attention as such. At one facility, we know there is an informal motto that runs along the lines of: “they’re all a group of fish, but every fish is an individual” (interview with Eugenie, aquarium facility manager, 8 February 2018). However, at the same time, it is acknowledged that this requires effort to sustain, and successful and lasting individualisation is the exception, not the rule (see also [95]). For all these and other reasons—some of which will be explored in more detail below—it is common to hear the argument that the apparent lack of social or ethical concerns associated with the use of fish in experiments is *in itself* one of the advantages of using zebrafish-based model systems, e.g., [20] (p. 407–408). Fish in general, but zebrafish specifically, are indeed frequently viewed as the “easier ethical option”, as one participant in our study put it (interview with Helen, representative of an animal welfare organization, 9 January 2019). In sum, while similar things may be said about other fish species, there are good reasons to pay special attention to zebrafish.

## 3. Results and Discussion

Analyses of our interview data suggest the presence of three especially significant social norms or discourses about fish welfare in the laboratory context. For summary purposes, we have labelled these “knowledge and consensus”, “attitudes and experiences”, and “institutional support and capacity”. Each of these narratives is internally diverse in terms of the individual opinions expressed, as well as overlapping and mutually reinforcing. Key themes included in the discussions which follow include: (structural) enrichment, fish, regulatory attitudes with respect to fish and the public; views about fish, embryos and larvae from within the aquarium and the size and composition of the zebrafish community

### 3.1. Knowledge and Consensus

Appendix A of the European Convention for the Protection of Vertebrate Animals used for Experimental and other Scientific Purposes (ETS123) provides key guidelines for the accommodation and care of animals used in science across Europe. Speaking about the challenge of managing a number of expert working groups convened by Council of Europe during the process of revising Appendix A in the early 2000s, an ex-UK Home Office Inspector told us “*[It] was like herding cats—they would not agree on anything*” (interview with Colin, ex-Home Office Inspector, 26 June 2019). In his experience, the field of fish welfare has been characterised by a lot of disagreement, often underpinned by insufficient knowledge of fish/zebrafish welfare science and of the basic biology that informs it, at least in comparison to the knowledge of the other major laboratory animal species. Similarly, our research suggests that, amongst those involved in the worlds of zebrafish science, there is limited consensus on what best practice is in a number of important welfare and 3Rs-relevant areas. Debate rages on numerous topics, including stocking density, food and feeding regimes, methods of anesthesia, euthanasia, the need for analgesia, and the need for environmental enrichment, to name only a few. This is reflected, as Colin explained, in the relative paucity of official guidance available for fish at the EU level or the level of individual member states—even for zebrafish, which are the most studied and used species. This section therefore explores narratives about knowledge, consensus and disagreement, focusing on two different examples. Firstly, the question of environmental enrichment, and secondly variation in beliefs about the ability of fish to feel pain and suffer, both of which are clearly relevant to welfare generally and the 3Rs specifically.

#### 3.1.1. “Putting Things in Tanks”

There exists a division in the zebrafish community in the UK between those who are in favor of environmental enrichment, and those who raise concerns about it (see also [96] p.586). To be specific, some facility managers and technologists—experienced husbandry professionals, some with backgrounds in relevant scientific disciplines—express doubts about the benefits of structural enrichment: the addition of plastic plants, substrates and so forth so as to provide cover and stimulation for fish who otherwise live in barren, clear plastic tanks. Some suggested that structural enrichment can encourage abnormal behavior, but the most common issue raised was that the welfare benefits of structural enrichment were not very clear. Felix, an experienced facility manager with a background in research, suggested there was a fashion for “putting things in tanks” (interview with Felix, aquarium facility manager, 1 November 2018). Felix, and others whom we have spoken to who share his point of view, are far from dismissive of enrichment for fish in general but worry that a focus on structural enrichments is a distraction from the factors they think are really more important for fish welfare and should be the focus of attention. Felix terms these more important factors the “subtle enrichments, your lighting, your temperatures, your feeding, your flows, those are a much more valuable asset than a plastic plant within a tank”.

People like Felix worry that the evidence base about the value of such structural enrichments for zebrafish is weak. An ex-Home Office inspector comments:

*I think probably the biggest constraint is just actually the lack of good data and scientific knowledge about what an appropriate environment for the zebrafish might be. I think again there is quite a lot of anthropomorphic views on what a zebrafish actually requires. You put them into an empty tank and that must be bad for them so they then put in lots of substrate and weeds and various other things in as well, you know, but we don’t really know, I don’t know…*

[interview with Craig, ex-Home Office Inspector, 25 June 2019]

Another facility manager concurs, arguing the welfare benefits of this kind of enrichment are in her estimation “fairly unproven”:

*[W]e can’t make that much more progress I personally don’t feel unless we can really say this is what is good for them in terms of like environmental enrichment, do we want divers in there with bubbles, and why would we want that, where do they ever see that, or plastic plants, would they see that in the wild? Is it appropriate?*

[interview with Fae, aquarium facility manager, 27 February 2018]

No one, of course, is suggesting the use of plastic divers and shipwrecks in academic research aquariums. The point being made is that the desire for objects in tanks is largely a human one: it satisfies our humane and aesthetic demands, rather than (so the suggestion goes) the real needs of the animals (as far as we know). Hence it has been pursued in the absence of evidence about its benefits. Fae and others in her position do not disagree that it is possible to observe certain behavioral changes on the introduction of an object like a plastic plant or simulated substrate, for example, which suggest a preference for occupying enriched parts of the tank. It is the interpretation of what these observed behavioral changes might actually mean for fish welfare that is questioned.

These kinds of concerns were echoed by animal technologists who work closely with fish. For example, Frank noted:

*We can look at cortisone levels or whatever but you don’t really know if you’re actually helping them. Like with a plant, I mean on one hand you’re creating cover for them to hide in if they’re getting bullied or fighting, on the other hand, you’re creating something for someone to get territorial about and stressed about.*

[interview with Frank, senior animal technologist, 18 January 2018]

A Home Office inspector also noted that

*[I]n terms of enrichment, for instance, we don’t actually know largely what fish want. […] And I think that--, that’s a significant challenge to get over some of the hurdles and show people how it can happen.*

[interview with Gail, Home Office Inspector, 15 May 2019]

While Gail sees this lack of knowledge as a barrier to acceptance, this does not feature in her discourse as a reason for being cautious about advocating their uptake in aquariums. Evidently, people operate with different ideas about what a sufficient evidential bar is. This reflects divisions in the field of laboratory animal welfare more generally as to whether the intuitions and experiences of the practices and protocols developed by technologists in individual facilities offer a strong enough evidence base for novel enrichment practices [75]. In this context, it is, of course, possible to cleave too bio-physiological measures of “health” only, in which the psychological and emotional factors usually comprehended within the wider term “welfare” are excluded. [44]. However, the latter, more encompassing and holistic outlook certainly seems to motivate managers and technologists who go out of their way to provide structural enrichments when they can. Sometimes this can be a real labor of love. One establishment found it could not afford to buy plastic plants from a hobby shop, so developed a way of making “plants” by fashioning them from plastic bags and weighing these down with marbles. This took six months of soaking the bags in a light bleach solution to stop the plastic leaching substances that may interfere with scientific results, and careful and time-consuming handwork by staff members to shape the fronds and attach the weights [RM, Field Notes,11 January 2018]. 

Advocates of structural enrichment do cite published evidence in favor of their opinions. A paper suggesting that zebrafish express a preference for substrates by positioning themselves over photographs of gravel is particularly often cited in the UK (see [97]). Those already inclined to enrichment tend to find such evidence a better reason to act than others who are not. An animal welfare policy expert felt that these results clearly “show that they [zebrafish] benefit from environmental enrichment”, but implied that this evidence was ignored (interview with Helen, representative of animal welfare organization, 9 January 2019). Others object to this interpretation of the meaning of fish preference behaviors or report having been told of (the referecne to hearsay is deliberate here) statistical or methodological weaknesses in papers about enrichment, and explain that people they know—others in the field—have taken these as a basis for inaction. 

We would suggest that these differences cannot be understood by looking at the published scientific literature only. Technologists and facility managers are moreover also at pains to point out the practical and economic downsides of structural environmental enrichments. For example, they can obscure technologists’ view of the fish whilst performing mandatory health checks, slowing them down and potentially leaving them less time for other important husbandry and welfare issues. They may also gather dirt and become unhygienic, and of course, they cost money to purchase in the first place which may have previously been allocated elsewhere. 

Another factor shaping orientations on this topic seems to be an identification with and long-term exposure to the world of mammalian husbandry, and especially previous experience working with rodents. Amongst our participants, those who most clearly expressed skepticism towards structural enrichment (plants, houses, substrate or even images of substrate), tended to identify strongly as “fish people” first and foremost. They may, for example, have backgrounds in marine biology, aquaculture, or hobby aquaria, or simply have no or limited professional interaction with the world of rodent husbandry. In some cases, the facilities which we visited who do not enrich as a matter of course are geographically, socially and administratively separate from the biological service facilities which run mammalian animal units. They tend to see the need to put “things in tanks” as something imported from the world of “fluffies” (as the technologists at one facility called them), and often pushed by people with more knowledge of mice specifically than of fish. Fae again expresses the point: “we look to mammals”, she told us “and go oh yeah environmental enrichment, that’s structural things in tanks” (interview with Fae, aquarium facility manager, 27 February 2018). 

On the other hand, Fae herself recalled how 20 or 30 years ago, it was common to see mice and rat cages that were entirely devoid of structural enrichments, like many zebrafish tanks today. Thus, the experience with rodents gets overlaid onto fish, as though fish must, or should be, on the same trajectory. In this case, “things in tanks” follow from being used to seeing “things in cages”. Evelyn, who is has extensive experience in all manner of mammalian husbandry, including running rat and mouse houses, and who takes pride in the compliment that her aquarium is run “like a mouse unit”, told us that

*if the mice were in the same situation 20 years ago [as the fish are today] they were just mice, but now like we have to provide enrichment, we have to provide certain bedding and nesting materials, we have to do this, we have to do that, and at some point or other, maybe not in my time, but the fish will have the same rights [laughs] somewhere along the line.*

[interview with Evelyn, aquarium facility manager, 18 January 2018]

Thus, knowledge of the welfare trajectory of mice is actually an explicit motivation for pursuing innovations, including enrichment, for fish in some cases. To be clear, the point is not that some of our research participants objected to better or more complex enrichments. Rather, they expressed skepticism about whether structural enrichments specifically have positive welfare effects that outweigh their downsides in different circumstances. Notably, it seems that this doubt is likely to be spiced with concerns about the source of advocacy for structural forms of environmental enrichment, including the belief that this is an ideology that is imported, without due consideration to context and species differences, from the world of rodent husbandry. Such views are connected to *identity* as well as to evaluations of *evidence*.

The matter became more acute for some participants when they perceived pressure to adopt structural enrichments to be coming from regulatory authorities, the most visible face of which are the UK Home Office Inspectors (HOI). Felix, for example, stated that, in his view, it was the Home Office who starting pressing for enrichment for fish “because that’s what they did for rodents”. Cynically, he concluded that “[I] could have solved the majority of my problems [related to facility inspections] if I had just had a plastic plant in the tank” (interview with Felix, animal facility manager, 1 November 2018). Another facility manager we spoke to, a keen proponent of enrichment for all kinds of animal, lamented the fact that, in his opinion some researchers do actually choose to enrich as a kind of virtue signaling to outsiders, especially the Home Office, not because they actually care much about what it might mean for animal welfare (RM, Field Notes, 16 August 2018).

The objection to “putting things in tanks” is thus sociological as well as scientific: those making the argument draw not only on scientific evidence but on their understandings of the views of outsiders to the fish world and their relationship to authority figures, as well as more pragmatic material and economic factors. We would suggest that similar combinations of factors are present in the instances of a variety of other disagreements associated with fish welfare in the aquarium. It is also important to note that everyone would welcome more research into the use of enrichments specifically and the biology of fish welfare generally. However, it would appear—and this is an issue demanding more research—it is equally important to achieving a sense of agreement on the underlying framework for deciding on what good welfare is and how it should be assessed. Furthermore, as we’ve suggested, *where* welfare recommendations are produced or come from (by scientists, by technologists or by particular groups or individuals, for example) and who they are promoted by (aquarists, “mouse people” or an HOI and so forth) can be very important, over and above the recommendations themselves, in determining their reception by the laboratory animal community. 

#### 3.1.2. Pain and Analgesia

Although the view is not universal, for many people who work with fish, fish welfare generally and the 3Rs specifically only have meaning on the assumption that these animals are sentient beings, feel pain and suffer as a consequence [88,98]. This intuition is reinforced by the fact that the law in the UK and the European Union effectively assumes that they are sentient and certainly that they feel pain. As such, the ongoing and high-profile debate about whether or not fish have, as a matter of scientific fact, the capacity to feel pain and suffer [35,99,100] has limited direct influence on the implementation and development of 3Rs-orientated welfare initiatives targeted at fish. In the day-to-day running of laboratory animal facilities, good animal welfare is a matter of complying with regulation, not challenging the epistemic or ethical assumptions of the law with respect to the possibility of emotional and subjective experience in fish. 

Nevertheless, the fact that there is a continuing controversy about fish pain might have a variety of more-or-less indirect effects that are relevant to understanding barriers to implementing the 3Rs with fish. Almost everyone we spoke to about this issue expressed varying degrees of uncertainty about whether fish actually feel pain, what this means to them, and whether humans will ever be in a position to know much about this. Given the oft-remarked phenomenon of “sentientism” (not to mention “speciesism”), this is unsurprising [101]. In some cases, though, opinions on the subject were connected directly to the scholarly debate. For example, one researcher who takes a negative view of the issue argued:

*“What I want to say is, erm, I think it is a difficult subject because until you really know, you can’t estimate what an animal perceives or what it doesn’t perceive. But what all the research shows at the moment is that you [the fish] do not have the higher brain structures required to perceive pain.*

[interview with Hanna, researcher, 27 November 2018]

In other cases, it was based more on personal intuition and belief. Referring to the behavior of post-operative fish, Evelyn said:

*They [the fish] act like everything is fine, but there is always a nagging doubt in the back of my head, there always will be. My dad was a fisherman, you know, and you can’t tell me that having a hook through your lip is not going to be painful. Can they [the fish] feel it? I don’t know.*

[interview with Evelyn, animal facility manager, 18 January 2018]

It is hard of course in any case to attribute the causes of particular actions or lack thereof specifically to beliefs about fish pain. However, there are a number of specific areas where such beliefs are more likely to shape action and influence debate in the field. 

The foremost example of this is probably the use (or lack thereof) of analgesia. Some of our participants pointed out that it is a default legal requirement to administer pain relief for all protected animals when appropriate, yet in the case of fish there was no standard analgesic authorized for use, nor indeed is analgesia use as widely practiced as it could be. There are many reasons that the use of analgesia following procedures on small fish like zebrafish may be problematic. Many of these refer to basic problems of a lack of evidence and/or consensus. Problems include a need to better understand the trade-offs between analgesia and other welfare concerns. For example, does the benefit of analgesia for a social species like a zebrafish outweigh the benefits of remaining in group housing, since isolation is usually necessary to administer it, and in what circumstances? They also include limited knowledge of the potential confounding variables that analgesic agents can introduce into experimental outcomes, a lack of knowledge of the pharmacological effects of different analgesic agents on different species, as well as problems connected with how to recognise and assess the effectiveness of these agents in these animals [102,103]. 

Nevertheless, more than one veterinarian has proposed that the existence of controversy on the subject of fish pain could be an underlying factor explaining the unwillingness amongst those responsible to implement analgesia protocols [104]. In the opinion of Schroeder and Mocho [105] (p.36), moreover, there is a danger that prospective applicants for licenses downplay evidence suggesting that fish do feel pain in favor of emphasising that it has not been conclusively shown that they do, and interpret the latter “as ‘carte blanche’ to avoid the use of analgesics altogether”. While much more fine-grained evidence needs to be gathered in order to understand resistance to, or at least slowness of spread, of analgesic protocols at the facility level, there are reasonable grounds for considering the fish pain controversy to be a contributory factor. While pain itself could be seen as introducing confounding effects, in one case we are aware of, permission not to administer analgesia following invasive surgery was granted for a combination of reasons, both scientific (related to the introduction of confounding effects) and welfare based, including the production of published arguments suggesting that fish are unlikely to experience the emotional effects of pain of the sort associated with higher and forebrain structures in mammals (interview with Hanna, researcher; RM Field Notes, 11 January 2018). So, plausibly, it is at least something which could tip the balance against analgesia in tie-breaker situations. Again here it is not only the scientific evidence which is shaping decisions about fish welfare, but how that evidence (or the lack thereof) is selectively deployed in decision making processes, with individuals most drawn to the evidence they believe supports their case, as has long been observed by science studies scholars in a range of fields of research, e.g., [106].

While the debates over analgesia use offer an example of the fish-pain controversy potentially shaping welfare practice, the most important effects of the ongoing debate on fish pain are likely to be more diffuse, influencing attitudes and priorities in subtle ways. In particular, there is the possibility that uncertainty about the nature of fish sentience gets shifted into plausible but unsubstantiated beliefs about fish’s *relative* lack of sentience in comparison to other vertebrates in some kind of a putative scale of sentience for which there is little or no objective basis. While regulations sensitive to the recognition of degrees of sentience may one day be possible and desirable [107], as it is such views about differences amongst vertebrates are likely to be informed by outdated ideas about the phylogenetic scale [91], as well as more arbitrary and sentimental ideas about what people believe is acceptable to do to different categories of animal [6,9,108]. A Home Office Inspector we interviewed tried to pick her way through this complex terrain:

*So there’s been huge arguments over those 20 years about are fish sentient at all? […] And I’ve always taken the presumption, well it’s in our law, that they wouldn’t be protected if they weren’t sentient--, if we didn’t believe they were sentient [they wouldn’t be there] and therefore we should be doing the best that we can for them. But equally they are a fantastic model as a replacement because, as far as we know, they are less sentient than other species, but we don’t know. So we would still suggest that it is better to move into zebrafish than to use mice, and I say that with some hesitancy […] most people in society I think would be more comfortable with fish being a replacement for mice.*

[interview with Gail, Home Office Inspector, 15 May 2019]

In this passage, it is very clear how beliefs about what the public feel about the use of different animals fills the gap opened up by the admission of fundamental uncertainty about the nature of fish sentience and experience. This movement is common in our experience. The concept of “societal sentience” has been proposed to understand such situations [109]. This refers to how people, especially policy makers, imagine what the public feels about animals—i.e., it is the feelings (sentience) of people that are in focus here, more than animals. In this context, relationships and attitudes to animals like fish, as well as beliefs about the extent to which those attitudes are shared with a wider ‘socially sentient’ public, can become extremely important in mediating decisions about their use and, by extension the urgency and relevance of 3Rs initiatives. This is the subject of the following section. 

### 3.2. Attitudes and Experiences

When there is an acknowledged lack of scientific consensus on issues that are of community and policy relevance, the values and perspectives held by collective and individual stakeholders can play a key role in shaping policy decisions and practices [110]. This section explores attitudes towards fish and the 3Rs held by those working in research settings and the factors shaping them. It focuses on the influence of those involved in regulation, including policy documents and legislation, as well as those with first-hand experiences working with zebrafish. 

#### 3.2.1. Regulators and the Public

The legal framework that regulates animal research in the UK is remarkably un-speciesist. Fish are formally afforded the same protections as most other vertebrate animals. For example, the two most commonly used laboratory animals—fish and mice—have exactly the same status in legislation. The importance of this should not be underestimated—not only in legal terms, but in terms of the broader agenda it helps set and the message it sends to all who work in the field. However, this picture changes somewhat with a closer look at the legislation and especially its modes of implementation. What emerges is a sense of hierarchy. In the UK, some animals do have some additional protections consequent on their special status in human society (primates, cats, dogs and horses). This suggests a subtle gradient of “social acceptability” in terms of what is expected to be tolerated by the public [109] (p.683) and, consequently, a prioritisation of the interests of certain species above that others emerges.

In the UK, the Animals in Science Regulation Unit (ASRU), based in the Home Office, is responsible for regulating the operation of the Animals (Scientific Procedures) Act 1986 (ASPA). ASRU’s Inspectorate division plays a key role in interpreting and applying the law and developing policy. A central element of this is reviewing and approving project license applications (a license is required to perform regulated experimental procedures on regulated animals in the UK). As a part of applying for a license, prospective licensees are required by law to demonstrate they have considered the 3Rs in the development of their research program. To assist applicants in completing the necessary documentation, the Inspectorate produce an annotated license form. This guidance document suggests applicants justify that their chosen animal model is the most refined possible, asking as prompt: “Why can’t you use animals with a lower capacity to experience pain, suffering, distress or lasting harm, e.g., fish instead of mice?” [111] (p.22). (The document is called “ASPeL Project License Application Template—General License Under the Animals (Scientific Procedures) Act 1986”, version V 2.0 21/12/17 and is (at the time of writing) still available via the Home Office website. Previous versions contained similar advice.) It is hard to assess the specific effects of this kind of “official” advice, but it is likely to have proven important in the past in promulgating the idea that the use of fish versus mice (or other mammals) is a kind of refinement or “relative replacement” in itself. For example, one grant awarded by the UK’s main 3Rs funding body, the NC3Rs, explicitly described the use of zebrafish as “a great opportunity for reduction of the use of higher order vertebrate species thereby reducing animal suffering” as its central 3Rs justification see [112]. This suggests embedded social and cultural assumptions about the sentience of fish and mice that are hard to justify purely in scientific terms. Implicitly or explicitly viewing the use of fish versus mice in this way sits awkwardly with the formal equality articulated in the definition of protected animal in legislation. It actually undermines what has been years of effort by Inspectors and others in the UK to elevate the status of fish and promote their welfare—and indeed recent advice has clearly moved away from this. 

The management of risk is a related important way in which representations of the public’s putative attitude towards fish may be made relevant to regulators. In our interviews, HOIs themselves talk a lot about risk assessment and risk management, particularly in terms of the allocation of Inspector resources and site inspections. A risk-based approach has become increasingly central and formalised in the wake of tightening budgets in the last decade. Risk in this context refers to a number of things, especially risk of non-compliance with the law and the presence of new species and use of novel procedures at a facility [113] (pp.22–23). But what could be described as “societal concerns” also emerged in our research, informing these sources of risk and adding their own dimension (see also [114] p.16). This includes concerns about the possibility of overt public outcries over the use or mistreatment of animals in research, and we suggest that in practice this is often interpreted as political or reputational risk to the Minister. For example, Craig, reflecting on the acceleration of a risk-based management approach at the Home Office, told us that in his opinion “the whole thing that actually drives ASPA [legislation] I think is essentially public perception” (interview with Craig, ex-Home Office Inspector, 25 June 2019). Thus, work involving specially protected species for which the public has great sympathy, such as with nonhuman primates, is typically viewed as especially high risk. Heather also noted how “public perception and risk” are a part of the calculation which informs how Inspectors allocate their attention:

*You know, so obviously, the feeling is that the public would prefer, if you like, primates to be inspected more frequently and are maybe less concerned about mice being [inspected].*

[interview with Heather, ex-Home Office Inspector, 17 January 2019]

This is clearly connected to ideas about species hierarchies, albeit in contextually specific ways.

Craig and Gail also referred to conservation work involving badgers as an example of an especially high-risk research program because badgers are believed to command a great deal of public attention and have been widely politicised in the UK (see [115]). In contrast, Gail says, “there are people who are doing conservation work on species that perhaps are not so high in the public consciousness and so the relative risk for the Minister then is lower” (interview with Gail, Home Office Inspector, 15 May 2019). Things which are likely to scandalise the Secretary of State are, of course, breaches in legal compliance in terms of animal welfare law, and harms being caused which are indefensible in the face of limited benefits. Thus, animal welfare and the management of (political) risk are not incompatible goals, but they are not necessary always perfectly aligned either. Zebrafish per se cannot be said to rank as a low priority in all circumstances—this would be too much of a generalisation and a lot of effort has gone into trying to raise the profile of zebrafish welfare at the Home Office and beyond. However, given what has been said about the perception of fish, their representation in the regulatory discourse, and widespread assumptions about what the public feel about fish, it is perhaps not unreasonable to suggest that they might occasionally fall between the cracks created when the different prerogatives of risk assessment are misaligned. This could contribute in turn to the effective, though unintentional, marginalisation of fish and a reduced likelihood of their being prioritised as candidates for investment in welfare interventions. 

The relative marginalisation of fish is also evident in the materials of organised campaign groups, who are capable of aggregating and directing diffuse and ill-formed public sentiment, and who are an important intermediary in shaping the perspectives of those who make and enforce and policy [116]. A review of the homepages of relevant organisations in Britain suggests that they do not consider fish an important focus for their campaigns. The websites of the main groups campaigning for the abolition of animal research in the UK (Cruelty Free International, National Anti-Vivisection Society, Animal Free Research UK) feature a total of zero images of fish on their homepages, though primates, dogs and rabbits are well represented. The RSPCA’s Laboratory Animal’s webpage, despite the organisation’s important role in disseminating husbandry and welfare standards [71] and raising awareness of fish welfare in UK labs, likewise featured no images of fish at the time of writing. Turning to the homepages of UK-based organisations specifically focused on funding 3Rs initiatives and/or the development of alternatives to the use of animals in science (FRAME, NC3Rs, Lord Dowding Fund), we find a total of one fish-related image out of a total of 17 representations of animals displayed at any one time. Indeed, fish are not the “poster critters” of animal research generally: homepages of the major “industry bodies” (Institute of Animal Technology, Laboratory Animal Science Association, Laboratory Animal Veterinary Association) feature zero images of fish out of total of 15 representations of animals. (This analyses was performed on 20/08/2019. All identifiable representations of animals were counted, including organizational logos. Addresses for the relevant websites are as follows: National Anti-Vivisection Society: http://www.navs.org.uk/home/; Animal Free Research UK: https://www.animalfreeresearchuk.org/; Cruelty Free International (previously BUAV): https://www.crueltyfreeinternational.org/; RSPCA Laboratory animals: https://www.rspca.org.uk/adviceandwelfare/laboratory; FRAME: https://frame.org.uk/; Lord Dowding Fund: http://www.ldf.org.uk/research/; NC3Rs: https://www.nc3rs.org.uk/; LASA: http://www.lasa.co.uk/; LAVA: https://www.lava.uk.net/index.php?sid=2dc936c14ca15e85e7d76b7d6b23092a; IAT: https://www.iat.org.uk/. The National Anti-Vivisection Society and Lord Dowding Fund websites feature a new stock image in their banners each time their pages are refreshed. Out of a total of at least 18 unique images registered, none feature fish.) Thus, if the unofficial status of fish is at least partially a function of the perception of the Inspectorate with respect to societal concerns and political risk, it is arguably a reasonably well-grounded one.

#### 3.2.2. Relating to Fish in the Aquarium

The views of the “general public” are thus important (see, e.g., [18]), but what is perhaps most critical in the light of our discussion is appreciation of how the public is imagined by policy makers [109]. This imaginary in turn shapes the priorities of regulators with implications for the scrutiny and prioritization of 3Rs efforts. This could be viewed as a “top-down” influence on the implementation of the 3Rs for fish. Complementing this insight, we turn attention in this section to look in more detail at some of the views about fish held by technologists, aquarium managers and veterinarians, because these are also central to the development and application of the 3Rs in situ [75], or from the “bottom-up”.

In our experience, it is very common for people who work with fish on a daily basis to object to what they see as the semi-official neglect of fish and the tendency to view the use of fish as in itself a kind of refinement or even replacement. These attitudes are often accompanied by a desire to advocate for fish and see them treated equally with other animal denizens of the lab:

*So, I think this idea that fish are some sort of replacement, I don’t think it’s right because we’ve decided to protect these animals so they should all be treated equally.*

[interview with Fiona, Named Veterinary Surgeon, 8 February 2018]

*They should have the same rights as everything else, and it might be just a fish, but going back a very long time someone told me that it was just a monkey… So you know, there should be no difference in my--, I know a monkey is a monkey and intelligent, but they’re in this building looking at us to be their eyes, ears and voice and protect them, there should be no difference whether it’s a fruit fly or a fish or a monkey or a pig or a mouse, whatever.*

[interview with Evelyn, aquarium facility manager, 18 January 2018]

At one animal facility, we observed how a poster on a corridor wall advertising aspects of the European Directive (2010/63) on the use of animals in research was decorated with images of small furry mammals. Irritated by the absence of a representation of fish, aquarium staff had stuck pictures of fish over them [RM Field Notes, 11 January 2018, Figure 1]. 

It is also common for people who work regularly with fish to say that they see fish and other animals as equals, but that they are aware of people who do not:

*[For me a fish is] still a living being so I don’t see it as being different myself. But I think a lot of people feel differently.*

[interview with Francis, researcher, 20 April 2018]

At the same time, however, people who work with fish will also often admit that they themselves do not feel the same way about fish as they do about other animals, especially mammals. Asked whether she empathised with her fish, Erica demurred with some difficulty:

*I think that [the word empathy] might be too strong. But definitely in that direction. Yeah, it’s because their faces are different [laughs], so you can’t really empathise with something that looks different from you, I think. Not that I’m saying that’s the right thing, but--,*

[interview with Erica, senior animal technologist, 23 April 2018]

Despite being, as we have seen, a very enthusiastic champion of the “equal rights” of fish and advocate of laboratory animal equality, Evelyn admits that she finds working with fish emotionally less engaging. Fish are more difficult to attach to than animals like primates, pigs, sheep, rabbits and rats [interview with Evelyn, aquarium facility manager, 18 January 2018]. Grant, an experienced fish researcher and keen aquarium hobbyist, noted:

*[…] from a personal point of view you can fairly well guess I care about my fish, and that lights me up. […Yet] I still feel more comfortable that we would use a fish rather than a mouse any day of the week. Even the smartest fish. That step into mammals--, […] is a difficult thing to deal with.*

[interview with Grant, researcher, 6 February 2018]

Gideon, another zebrafish user, declaimed the “double standard”, as he sees it, that gets applied to fish, but then noted that he also understands why the double standard exists because he feels it himself, and speculates on the causes:

*Yeah, less emotional attachment. It’s undeniable, it’s not the same. […] I don’t know, maybe because it’s sushi.*

[interview with Gideon, researcher, 9 October 2018]

Frank, reflecting a very common theme amongst aquarists, noted that if he “had to cull a pig or a dog or a cat, I wouldn’t be in the job”, and explained that:

*I’d rather work with fish because you don’t get the attachment that you would with mice. Maybe I’m the other way. I try not to be, but I am quite sort of, I can be anthropomorphic. I know that you can’t reflect your emotions onto them but it’s hard not to do so. In that sense I don’t have that relationship with the fish. I take them seriously and I care seriously and I want them to be healthy, but it wouldn’t keep me up at night, if I had to cull some fish at the end of the day it wouldn’t keep me up at night.*

[interview with Frank, senior animal technologist, 18 January 2018]

There are of course many reasons why fish generally and zebrafish specifically engender this kind of ambivalence, even amongst people who know them best and attend to them often on a daily basis. As discussed above, these include their small size, their relatively short lives and high reproductive rates, and the often very large numbers in which they are kept. All of these factors militate against humans forming lasting bonds with them as individuals. Then there are other specific biological and ecological characteristics of fish: their lack of “face” and “voice” with familiar interactional and emotional cues [66]. They lack what has been termed “nonhuman charisma” [117], an ascribed property of some animals that has been credited, in the context of animal conservation, with generating social interest and species–specific knowledge bases which in turn forms the basis for decisions on policy and funding priorities. In this sense, nonhuman charisma leads to what could be thought of as differing degrees of “political” influence for different animal taxa. 

In the case of aquarium fish, the water adds a further element of detachment; while wild fish are even more remote, it’s still the case that even when in the same room as us, captive fish live visibly separate lives from our own, behind glass and in a different element.

*Because they’re in glass tanks and they’re very separate you don’t kind of get that interaction quite the same as you would with a smaller mammal. [Consequently], it’s easier to kind of detach yourself a little bit emotionally from that fish.*

[interview with Gemma, senior animal technologist, 8 February 2018]

Such themes of perceived psychic distance are very common in discussions with technologists and others who work with zebrafish. For Fae, though, this results in a regrettable state of affairs. She argued that people’s ability to relate or attach to animals plays too big a role in driving priorities, to the detriment of fish welfare:

*And I think this is what it is, I mean this is what I find annoying at times, it’s not really about the fish it’s what people can relate to and what people believe, and you know this is why we have these massive variations in welfare with fish because people just don’t get it and like they’re not thinking--, you know, if they can’t relate to it themselves I think it’s much harder.*

[Interview with Fae, aquarium facility manager, 27 February 2018]

It is difficult to connect these kinds of attitudes directly to the situation of the 3Rs. Harboring the kinds of conflicted emotions that we have been discussing of course does not preclude one from being active in pursuing 3Rs initiatives because people can be inconsistent and motivated by many different and competing prerogatives at once. But areas where these kinds of feelings amongst scientists and technicians towards fish do seem particularly likely to influence their actions or priorities, however, include the assessment of welfare and especially severity. Feeling emotionally and thus morally distant from fish in their alien habitats could conceivably compound the practitioners struggle to recognise, evaluate or correctly rank relevant signs of ill welfare or suffering.

### 3.3. Institutional Support and Capacity

The challenges of a contested evidence base, combined with general sense amongst both general “public” and those working with laboratory animals who find it “hard to care” about fish are compounded by (and arguably compound) the challenges that are experienced in mobilising institutional support for 3Rs initiatives. It is obvious that the kind and degree of institutional and economic support for fish-focused 3Rs initiatives are crucial to their success. A great deal could be said on this point, though much of this would apply to barriers to the development of the 3Rs for all species, not just fish or zebrafish. We focus here on only one main point with the claim that, as important as zebrafish are as a model organism, the size of the scientific and technical community it supports are still significantly smaller than the mouse community, and this means that it often does not possess the diversity of functions necessary to identify problems and credibly take forward solutions to them. In his observations, however, ex-HOI Colin, who has a lot of experience across the European Union as well as the UK, made this point most perspicuously. While gesturing towards the issue of funding, he explained that the problem is not simply that there was not enough of it, but that, in comparison to rodents, there was not yet within in the zebrafish world sufficient capacity to compete for it on even terms. Thus, Colin described a relative absence of what he called a “welfare support group” comprised of “vets, technicians [technologists], and welfare scientists” analogous to that which exists for what he called “the furries”. As he explained:

*[fish-directed 3Rs research] is not sexy enough I don’t think and there’s not enough people involved to actually--, you know, because it’s difficult for technicians [technologists] to go to Wellcome [funders of biomedical science] and say, “Could I have a pot of money?” or even NC3Rs [National Centre for the 3Rs], whereas there’s a lot of people out there who’ve done animal welfare degrees or whatever and are interested in furries or whatever, and they know they’ll get funding.*

[interview with Colin, ex-Home Office Inspector, 26 June 2019]

And, he continued by pointing out that

*[…] the other issue with fish people, the fish scientists are--, zebrafish scientists tend to be totally focused on zebrafish and the science, they’re not all that interested in welfare, they’re not behavioural type people, whereas in the furry world you’ve got behavioural type scientists who are interested in [welfare and the 3Rs].*

[interview with Colin, ex-Home Office Inspector, 26 June 2019]

This is, of course, just the impression of one experienced observer, but it suggests an important point. Namely, that the diversity of available skills, interests and concomitant credibility is a function to some extent of the size of the extended community of practice. If this community is small, this represents an important sociological constraint on the development of new 3Rs interventions.

In our experience, many 3Rs-relevant welfare and husbandry initiatives are, in fact, driven “from the bottom up” by technologists and aquarium facility managers, not only academic research scientists. In the UK, centers of excellence have emerged around some, usually sizable aquariums managed by motivated individuals, though there is no particular pattern to this: in some cases, these individuals have backgrounds in general animal management or the management of rodents in particular but have become over time become leaders in the field of fish husbandry; in other cases, managers have backgrounds in aquatic biology, fish behavior or indeed biomedical research, and have moved into management. Many such initiatives are very local and small scale. The development of “DIY” environmental enrichment (see above) falls in this category, as does the move by one facility to introduce spirulina as an additive to fish diet (this has the effect of enhancing the lateral pink streak often expressed by male zebrafish. As a consequence, lab users are able to easily and reliably identify the sexes visually, instead of needing to anaesthetize them for closer examination as had been done routinely previously by some lab members). Other initiatives may begin life on the aquarium floor, but expand outwards: for example, the first Body Condition Scoring System for zebrafish was developed by staff at University College London’s zebrafish aquarium [118]. Initiatives such as the Zebrafish Health and Welfare Glossary, which promotes a standardised approached to welfare evaluation and nomenclature, have also been developed and primarily promoted by technical staff [119].

Local, technologist-led 3Rs initiatives are likely to vary considerably in the degree to which they are supported—in all senses of the word—by academic scientists and the wider bureaucratic, professional and institutional structures in which they emerge and to which they relate. While those who initiate and become involved in such efforts may have different or mixed motivations—from personal desire to change the lives of animals for the better, to a sense of professional responsibility or career ambition—it should be noted that in doing so they are liable sometimes to go beyond the proverbial “call of duty”, and there are consequently limits as to what can expected in terms of uptake and scale.

Academic researchers, of course, are not absent from this picture. Many 3Rs-geared ambitions for zebrafish would be impossible without specialist scientific expertise. In the UK, for example, Lynne Sneddon and colleagues have published influential data from numerous experiments focused explicitly on the possibility of deriving 3Rs interventions from them, for example, in areas such as analgesia research [81,120,121,122,123], enrichment [97] and automated welfare monitoring [124]. Academic researchers have also led in the development of protocols with direct 3Rs implications, for instance, concerning the genotyping of zebrafish by means of fin clips on very young larvae (3dpf) [125]. Indeed, looking at the database of funded research from the premier source of 3Rs funding in the UK—the NC3Rs—we find as of 2 December 2019 that with one exception, all funded zebrafish-based projects have established research scientists listed as their principal investigators. (See NC3Rs “Our Science” search results for the keyword “zebrafish”. The search was restricted to all kinds of grant and excluded training see shorturl.at/qL489. The exception is a veterinarian fronting a project investigating behavioral and physiological responses to fin clipping). This reflects the nature of applications received, and it is of course correct that awards for projects requiring detailed knowledge of scientific design are headed by those competent in this area, and is a reflection of active involvement by academics—notably, though, a large proportion of these awards focused on developing <5dpf embryos models, not on the benefits to fish as ends in themselves. It is of course possible that in some cases, technologists may be actively involved behind the scenes in some cases. Nevertheless, this should be set against Colin’s contention that those often most motivated to get involved with 3Rs work that benefits zebrafish do not have the means or credibility (including knowledge of research design, for example) to get the most desirable kinds of support for their work. Indeed, some may find themselves unable to apply for certain funding streams because of the non-academic classification of their roles and career trajectory, whatever their competence as scientists.

There seems certainly to be a niche or gap to be bridged between the local and less “science-heavy” 3Rs initiatives and those requiring special expertise in research design and data analyses, as well as specialist and expensive technologies. Developing and validating replacement models, for example, tends to be very “science-heavy”. Speaking of the importance of collaboration and capacity building in this area, one of our informants also described the need for what he called the “dovetailing” of interests, in particular, finding ways of bringing the scientific nous and technologies of academic researchers to bear on husbandry related problems in ways which could benefit everyone; scientists, technologists and fish. For example, the use of fluorescent markers of neuronal activity, as routinely done in many labs, could help to answer basic issues related to husbandry and welfare, such as the identification of appropriate endpoints (interview with Farol, aquarium facility manager, 21 March 2018). Efforts in this direction, however, face at least two general problems. Firstly, there is the problem of a lack of incentives for academics on a conventional scientific career path, given the lower status of such questions in the hierarchies embedded in the scientific reward system, and the relative ghettoisation of 3Rs and animal welfare science work generally. Secondly, the social stigma we have discussed that apparently continues to position fish as a means of achieving reductions or refinements, rather than as a focus for receiving 3Rs benefits. For Colin, at least, it is worth noting that an underlying reason for 3Rs/fish welfare research not being considered “sexy enough” (see above), is the attitude that “they’re only fish”, and thus do not warrant the attention (interview with Colin, ex-Home Office Inspector, 26 June 2019). These kinds of attitudes probably compound the basic problems of size and capacity suggested here.

## 4. Conclusions

While fish are rarely the “poster critters” of animal welfare campaigns, the welfare of aquatic species, in general, is increasingly becoming an object of social interest and concern, as well as scientific relevance. Moreover, given that ASPA makes no distinction between fish and other forms of vertebrate life in its definition of a protected animal, and that scientific opinion about the capacity of fish to suffer seriously is mounting, there is an ethical, regulatory and scientific remit for focusing on barriers to implementing and developing the 3Rs for fish. In this paper, we have shown how qualitative social science offers useful insights into the social drivers that could be influencing thinking, prioritisation and implementation of the 3Rs with respect to laboratory fish welfare.

Firstly, we highlighted the importance of narratives about knowledge, consensus and disagreement. In our examples, limited knowledge of what constitutes appropriate environmental enrichment for zebrafish and disagreements over the ability of fish to feel pain and suffer, can hamper the implementation of refinements, despite regulatory encouragement. Furthermore, an awareness of where knowledge about what constitutes “good welfare” is produced and who it is promoted by can be as important as the knowledge itself in shaping its reception and the consequent implementation (or not) of refinements. This is seen, for example, in the division between those with a lot of experience with mammals who are inclined towards “putting things in tanks” as they are used to seeing “things in cages”, and those who have worked mainly with fish and suspect other who suggest more subtle enrichments such as lighting regimes and water chemistry are more important. We also described how the existence of controversy on the subject of fish pain could be an underlying factor explaining the unwillingness amongst those responsible for implementing analgesia protocols, for example. We also proposed that there is a kind of scale of sentience, which ranks fish below other vertebrates and which shapes attitudes to fish welfare despite having little or no objective basis.

Secondly, we discussed how relationships and attitudes to fish, as well as beliefs about the extent to which those attitudes are shared with a wider ‘socially sentient’ public, may be important to mediating decisions about their use, their deployment as an alternative for other animals with the same legal status (such as mice) and, by extension, the urgency and relevance of 3Rs initiatives. For example, we noted how the apparently relatively low priority given to fish welfare amongst animal welfare and rights organisations is often linked to a perceived lack of broader public concern. Regulators may also follow suit, despite their best intentions and efforts. In this context, those who work with fish in laboratory settings often act as advocates for fish to be treated equally with other animal denizens of the lab. However, even within laboratory settings technologists, researchers and vets can struggle to relate to fish and find themselves questioning the extent to which they have internalized an image of fish as somehow less sentient and capable of suffering than mammals. This highlights the importance of a degree of self-awareness and reflexivity amongst those responsible for assessing fish welfare and implementing the 3Rs (already evident in the words of many of the those we spoke to) about how practices are shaped by social beliefs, experiences and values as well as scientific expertise.

Finally, we noted how more general trends towards a lack of investment and research interests in the 3Rs, recognised across the animal research community, are compounded by specific issues associated with the overall capacity of zebrafish community to engage successfully in 3Rs initiatives. In this context, we also presented the claim that zebrafish have not hitherto been perceived as “sexy enough” to attract the attention of enough credible experts in animal welfare science and animal behavior who are interested in pursuing 3Rs-related work. In our experience, moreover, many 3Rs-relevant welfare and husbandry initiatives are also driven “from the bottom up” by technologists and aquarium facility managers, not only by academic research scientists. This led to our informants highlighting the need for further collaboration and capacity building in this area, bringing the scientific knowledge and approaches of academic researchers to bear on husbandry related problems in particular, in partnership with motivated technical staff.

## Figures and Tables

**Figure 1:**
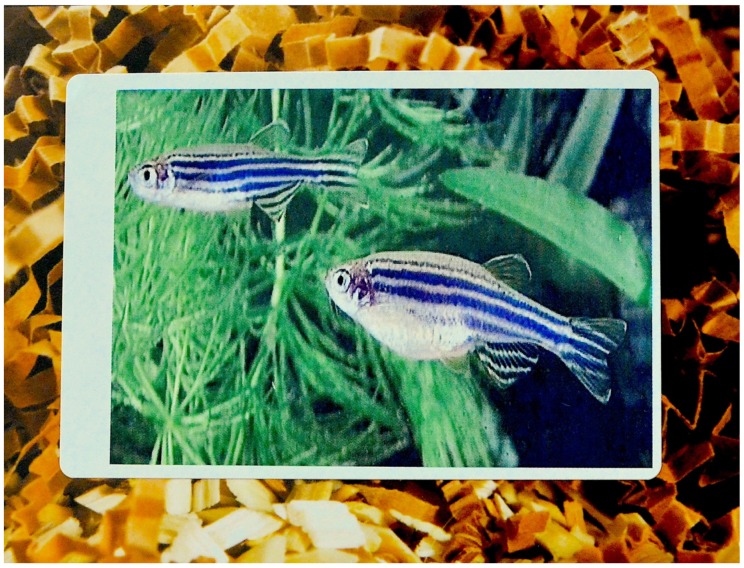
An image of zebrafish is pasted over an image of mouse on an education and training poster at a UK zebrafish aquarium facility (detail from poster). 29 October 2019. Photo credit: Reuben Message.

**Table 1:** Summary of main themes emerging from qualitative interviews.

1	Human-animal relations\Relating to fish
2	Animal research identities and group relations\Animal technologist-scientist relations
3	Protected life stages\The 5 days’ post-fertilization rule
4	Organisational and regulatory processes\Regulatory issues\3Rs
5	Welfare debates\Enrichment
6	Social relations, people and politics\Participants backgrounds
7	Organizational and regulatory processes\Regulatory issues\Home Office Inspectors
8	Model organisms\Model selection and uses
9	Model organisms\”Good Science” and reproducibility
10	Participants backgrounds\Early life experiences
11	Human-animal relations\Reading animal bodies
12	Rodent/fish comparisons\Comparing attitudes towards fish vs. rodents
13	Rodent/fish comparisons\Rodents as “models” for fish welfare
14	Aquarium practices\welfare checking, screening and quarantine procedures
15	Human-animal relations\Animal technologist-animal relations
16	Organisational and regulatory processes\Management issues\Training
17	Developing welfare systems\Standardisation practices
18	Protected life stages\Larval sentience
19	Aquarium practices\Feeding
20	Management issues\Economics
21	Regulatory issues\Opinions about ASPA (UK legislation)
22	Animal capacities\Fish pain
23	Management issues\Staffing
24	Purpose of research\Regulatory testing
25	Aquarium practices\Culling\Experience of culling

These themes represent the top 25 codes generated by the authors in the process of data analyses. They are reported in descending order, from most used to least frequently used. Codes and the themes they represent often overlap. The number of times a code is used can suggest the importance of the subject to both the speaker and analyst, but the frequency of use is not on its own a measure of importance or relevance to the present topic.
